# Supplementary material for: Learning curve and surgical outcome of robotic assisted colorectal surgery with ERAS program
Source: Sci Rep. 2022 Nov 29;12:20566. doi: 10.1038/s41598-022-24665-w (PMC9709162; doi:10.1038/s41598-022-24665-w)
Supplement: Supplementary file 2 — Supplementary Information 2. [file 41598_2022_24665_MOESM2_ESM.zip › RAL Raw data-Ñ[▒K/069/Patho_ñw╝╨░O▒Kñσ.pdf]

臺中榮民總醫院 臺中市臺灣大道四段1650號 病理部活體組織切片 臨床醫學實驗及病理診斷中心 檢驗(查)報告

[redacted]

Pathologic diagnosis:
1. Rectum, robotic abdominoperineal resection ---
Status post neoadjuvant concurrent chemoradiotherapy with ulcer and inflammation of anus. No residual tumor cell present. The bilateral cut ends and perirectal lymph nodes (0/4) are free of tumor.
2. IMA root, dissection --- Unremarkable lymph node (0/1).

Ancillary study for diagnosis:
Recut and IHC stain for AE1/AE3 are done on section C, no abnormal epithelial cell is highlighted..

Prognostic and predictive factor:
1. Tumor site: No residual tumor.
2. Tumor size: Not applicable due to no residual tumor.
3. Histologic type: Not applicable.
4. Histologic grade: Not applicable.
5. Tumor extension: Not applicable.
6. Surgical Margins:
a) Proximal Margin: Uninvolved by invasive carcinoma.
b) Distal Margin: Uninvolved by invasive carcinoma.
c) Radial or Mesenteric Margin: Uninvolved by invasive carcinoma.
7. Treatment effect: Present, no viable cancer cell (complete response, score 0).
8. Angiolymphatic invasion: Not identified.
9. Perineural invasion: Not identified.
10. Regional lymph nodes: Uninvolved (0/5).
11. Pathological TNM Stage: ypT0N0 (According to the eighth edition, American Joint Committee on Cancer Staging Guidelines for Tumors / 2017 CAP guideline).
12. TNM descriptors: y (Post-treatment).

Gross description:
The specimen consists of 1) a segment of colon, 13.8 cm in length and 3.5 cm in proximal cut end circumference, and perirectal soft tissue, with attached anal mucosa and skin, 8.7x5.5 cm. A gray tan ulcerative lesion, 1.2x1.1 cm, is noted, 1.2 cm above the anal verge and 2.3 cm from the anal skin margin. On cross section, the ulcerated lesion is indurated, 3 cm to the radial margin. Several polypoid nodules are also seen at the anal verge. The proximal cut end of colon and anal skin margin are free of tumor grossly. 2) IMA root, a piece of tan yellow soft tissue, 3.5x2.1x0.8 cm. Representative part for section: A) proximal cut end B-C) ulcerative lesion and deep margin D) anal skin margin E) polypoid nodules at the anal verge F) colon mucosa G) specimen 2 H-S) lymph nodes.
#T-59600_2 #M-09413_2 0 1105 SP0000
#T-C4560_2 #M-09183_2 0 1105 000000

已電子簽章

敬告：本單僅供醫師參考使用，不具任何申請效力！！
請病患妥為保管，若不慎遺失需自負個人資料外洩責任！
